# Computational modelling of scleral photocrosslinking: from rat to minipig to human

**DOI:** 10.1098/rsif.2024.0111

**Published:** 2024-07-31

**Authors:** Amy J. Wood-Yang, Brandon G. Gerberich, Mark R. Prausnitz

**Affiliations:** ^1^ School of Chemical and Biomolecular Engineering, Georgia Institute of Technology, Atlanta, GA 30332, USA; ^2^ Wallace H. Coulter Department of Biomedical Engineering, Georgia Institute of Technology and Emory University, Atlanta, GA 30332, USA

**Keywords:** computational modelling, glaucoma, ocular drug delivery, scleral photocrosslinking, photosensitizer

## Abstract

Selective scleral crosslinking has been proposed as a novel treatment to increase scleral stiffness to counteract biomechanical changes associated with glaucoma and high myopia. Scleral stiffening has been shown by transpupillary peripapillary scleral photocrosslinking in rats, where the photosensitizer, methylene blue (MB), was injected retrobulbarly and red light initiated crosslinking reactions with collagen. Here, we adapted a computational model previously developed to model this treatment in rat eyes to additionally model MB photocrosslinking in minipigs and humans. Increased tissue length and subsequent diffusion and light penetration limitations were found to be barriers to achieving the same extent of crosslinking as in rats. Per cent inspired O_2_, injected MB concentration and laser fluence were simultaneously varied to overcome these limitations and used to determine optimal combinations of treatment parameters in rats, minipigs and humans. Increasing these three treatment parameters simultaneously resulted in maximum crosslinking, except in rats, where the highest MB concentrations decreased crosslinking. Additionally, the kinetics and diffusion of photocrosslinking reaction intermediates and unproductive side products were modelled across space and time. The model provides a mechanistic understanding of MB photocrosslinking in scleral tissue and a basis for adapting and screening treatment parameters in larger animal models and, eventually, human eyes.

## 1. Introduction

Glaucoma affects approximately 80 million people and is expected to increase to over 100 million people worldwide by the year 2050 [1]. Moreover, glaucoma is the second-leading cause of irreversible blindness in the United States and has increased prevalence among racial minorities [2]. Increased intraocular pressure (IOP), retinal ganglion cell death and structural changes to the optic nerve head are some defining characteristics of glaucoma, which can lead to irreversible blindness [3]. Treatments include pharmacological and surgical interventions that are not curative and are usually needed for life [4].

Uncorrected myopia is the second most common cause of blindness worldwide, and myopic retinopathy causes irreversible blindness [5,6]. Axial elongation and subsequent biomechanical stretching on the posterior pole become worse as myopia progresses, and advanced myopia is a risk factor for glaucoma [6]. Current methods to prevent axial elongation in severe cases of high myopia include posterior scleral reinforcement, which is an invasive surgical procedure and can cause serious postoperative complications [5,6].

Scleral crosslinking using crosslinking agents (e.g. genipin and glyceraldehyde) has been proposed as a novel treatment for both glaucoma and myopia [7–9]. Scleral stiffness can be increased, which is hypothesized to reduce damage to the optic nerve head caused by mechanical deformation (i.e. cupping) from glaucoma [10–12] or can inhibit deformation of the shape of the globe that causes myopic axial elongation. Crosslinking agent is introduced into the sclera by ocular injection to crosslink tissue fibres, but this method of scleral stiffening has limitations, notably poor spatial control over crosslinking, because the location of crosslinking agent injection and subsequent diffusion determine which areas of tissue get crosslinked. Prior studies in mice found that whole-globe glyceraldehyde scleral crosslinking increased glaucomatous damage, by not selectively strengthening peripapillary sclera, where it was needed, and allowing scleral deformation in other parts of the eye in response to the IOP elevation [9].

Photocrosslinking could be an improvement on this ‘dark’ crosslinking approach, since light (visible or ultraviolet) is required to activate the photocrosslinking agent and has been studied for corneal and scleral crosslinking by riboflavin [13–15] and more recently, for scleral crosslinking by methylene blue (MB) [10]. Photocrosslinking allows for more selective control over the extent and tissue location of crosslinks than dark crosslinking, since crosslinking only occurs in areas of tissue exposed to the applied light.

Selective peripapillary scleral crosslinking is of interest for glaucoma treatment since this tissue undergoes more strain compared to anterior scleral tissue during IOP increase in glaucomatous patients [16,17]. It is our hypothesis that decreasing strain around the optic nerve by selective peripapillary scleral crosslinking could help prevent glaucomatous damage [10–12]. Transpupillary peripapillary scleral photocrosslinking treatment was previously demonstrated by retrobulbar injection of the photocrosslinking agent, MB, forming a reservoir of MB posterior to the sclera, from which MB diffuses to anterior tissues [10]. To achieve selective peripapillary photocrosslinking, visible light of 660 nm can be applied in an annular beam to prevent light exposure to the optic nerve head. The light causes photoexcitation of MB, which initiates photocrosslinking reaction schemes to cause crosslinking between collagen amino acids in the sclera. Additionally, selective equatorial scleral crosslinking by riboflavin and 370 nm light has been demonstrated in rabbits for the treatment of myopia [15]. However, ultraviolet A light has more energy than longer wavelength visible light, causing it to have a higher risk for ocular damage [18,19]. MB is also a more desirable photosensitizer over riboflavin owing to its absorbance in the phototherapeutic window of red and near-infrared light [20].

Desired outcomes for the MB photocrosslinking treatment include crosslinking the peripapillary sclera to an extent that allows for mechanical stiffening of the sclera. The MB photocrosslinking treatment in rats reduced scleral strain by approximately 50% [10], which corresponded to the 5 mM crosslink concentration projected by computational modelling [21]. To translate the treatment to larger animals like minipigs and eventually to humans, we need to understand how to use findings in smaller animals like rats to guide, predict and optimize scleral photocrosslinking in larger eyes with different tissue thicknesses and properties. Desired crosslinking outcomes are dependent on the ability of MB to diffuse in ocular tissues, ability of light to penetrate across and into the tissue, the availability of O_2_ in the tissue and the kinetics of crosslinking reactions [21].

**Figure 1.**
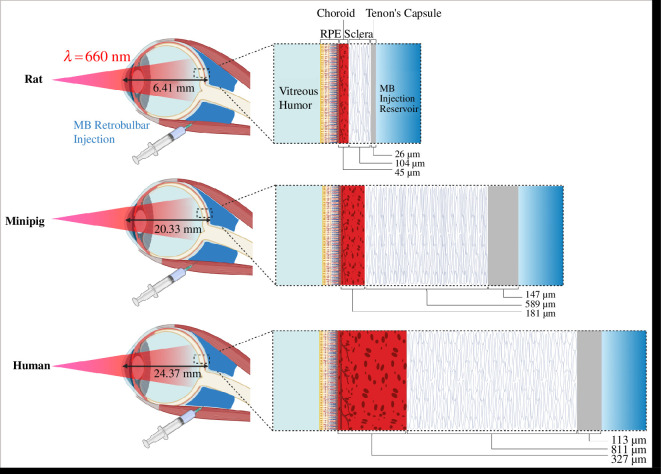
Comparison of tissue length scales in rat, minipig and human eyes (based on values in table 1). Tissue thicknesses represented to scale. Images generated using Biorender.com

Our lab previously developed a computational model to predict extent and distribution of transpupillary peripapillary photocrosslinking in rat sclera [21]. However, rat ocular tissue differs from larger animal models and human eyes, suggesting that photocrosslinking would require different treatment parameters in these species. Notably, rat eyes are much smaller than human eyes [22], whereas porcine eyes are more similar in size to humans [23] (figure 1). Also, in contrast to rats, porcine eyes have proportionally more scleral tissue, which is more representative of human eyes [24,25].

**Table 1.** Model parameters that vary among rats, minipigs and humans.

parameter (units)	description	rat [21]	minipig	human
*X*_RPE_ (μm)	RPE length	10	9^a^ [29]	26 [30]
*X*_Chor_ (μm)	choroid length	45	181 [26]	327^b^ [31]
*X*_Scl_ (μm)	sclera length	104	589^c^ [24]	811^d^ [25]
X_Ten_ (μm)	Tenon length	26^e^	147^e^	113 [32]
*X*_Inj_ (μm)	MB injection reservoir length	3100	308^f^	214^f^
*X*_Retro_ (μm)	distance from MB injection reservoir to retrobulbar tissue^g^	45	181	327
*C_0,_*_His, Scl_ (M)	initial AA concentration in sclera, adjusted for tissue density [21]	1.335 × 10^−2^	1.342 × 10^−2^	1.375 × 10^−2^
*C_0,_*_Met, Scl_ (M)		2.225 × 10^−2^	1.939 × 10^−2^	3.667 × 10^−2^
*C_0,_*_Trp, Scl_ (M)		8.90 × 10^−3^	8.95 × 10^−3^	4.58 × 10^−3^
*C_0,_*_Cys, Scl_ (M)		2.670 × 10^−2^	2.685 × 10^−2^	4.584 × 10^−2^
*C_0,_*_Tyr, Scl_ (M)		2.076 × 10^−2^	2.237 × 10^−2^	1.375 × 10^−2^
*C_0,_*_His, Chor_ (M)	initial AA concentration in choroid, adjusted for tissue density and luminal fraction [21]	6.46 × 10^−3^	6.49 × 10^−3^	6.44 × 10^−3^
*C_0,_*_Met, Chor_ (M)		1.076 × 10^−2^	9.38 × 10^−3^	1.716 × 10^−2^
*C_0,_*_Trp, Chor_ (M)		4.30 × 10^−3^	4.33 × 10^−3^	2.14 × 10^−3^
*C_0,_*_Cys, Chor_ (M)		1.29 × 10^−2^	1.30×10^−2^	2.15×10^−2^
*C_0,_*_Tyr, Chor_ (M)		1.004 × 10^−2^	1.082 × 10^−2^	6.44 × 10^−3^
*C_0, Mel, Chor_* (M)	initial melanin concentration in choroid, adjusted for tissue density^h^	3.05 × 10^−1^ [21]	3.05 × 10^−1^ [21]	6.00 × 10^−2^ [33]
*C_0,_*_Mel, Scl_ (M)	initial melanin concentration in sclera, adjusted for tissue density^h^	6.46 × 10^−2^ [21]	1.51 × 10^−2^ [21,34]	7.84 ×10^−2^ [34]
*C_0,_*_Mel_ *_,_*_RPE_ (M)	initial melanin concentration in RPE, adjusted for tissue density^h^	8.50 × 10^−2^ [21]	8.50 × 10^−2^ [21]	7.35 × 10^−2^ [33]
*C_0, O2,_*_Chor_ (M)	initial ^3^O_2_ concentration in choroid, 21% inspired O_2_	4.81 × 10^−5^ [21]	6.07 × 10^−5^ [35]	7.02 × 10^−5^ ^i^ [36]
*C_0, O2,_*_Res_ (M)	initial ^3^O_2_ concentration in reservoir, 21% inspired O_2_	2.18×10^−5^ [21]	2.18 × 10^−5j^	3.09 ×10^−5k^
*r*_Scl_ (g cm^−3^)	density of scleral tissue	1.077	1.077^l^	1.049 [40]
*r*_Chor_ (g cm^−3^)	density of choroidal tissue	1.063	1.063^l^	1.002 [40]

^a^ Estimated from histological staining of tissue samples.

^b^ Average between emmetropes and myopes.

^c^ The average of male and female minipig posterior scleral thicknesses was taken.

^d^ Average circumferential thickness was taken between slices 2 to 5.

^e^ There is no published value for Tenon length. Estimated to be 25% of sclera length.

^f^ See electronic supplementary material, §S1 for calculation corresponding to 100 µl injection volume.

^g^ Assumed distance for MB to be cleared from injection reservoir to retrobulbar tissue is the same as choroidal length.

^h^ Assumed 200 Da molecular weight of melanin for calculation in molar units.

^i^ Values reported in macaque monkeys were assumed to be a valid approximation for that in humans.

^j^ Assumed retrobulbar O_2_ partial pressures are similar to rat since choroidal O_2_ tensions in minipig are similar to those of rat.

^k^ O_2_ partial pressure at 21% inspired O_2_ in the retrobulbar muscle was assumed to be similar to that reported in other smooth muscles in humans (27 mm Hg) [37–39].

^l^ Assumed same value as rat.

Minipigs were chosen as an animal model to serve as a bridge between rats and humans, because minipigs and porcine eyes have similar sizes [26,27], but minipigs are easier to house for experimental studies compared to full-size pigs [28]. Differences in ocular geometry can impact the depth of light penetration into ocular tissues and the depth of MB diffusion from the injection reservoir. Additionally, tissue concentrations of melanin and amino acids differ among eyes of different species (table 1; electronic supplementary material, table S1), which can impact light transmission across ocular tissues and the extent of MB photocrosslinking. The aim of this study was to use computational modelling to predict and compare scleral photocrosslinking outcomes in rat, minipig and human eyes.

Using this model, we assessed the feasibility of performing scleral photocrosslinking at varied treatment conditions (laser fluence, per cent inspired O_2_ and injected MB concentration) in rat, minipig and human eyes. The model provides a basis for selecting treatment parameters in larger animal models and screening combinational effects of treatment parameters that can guide future animal studies that in turn guide studies in humans. Ultimately, the model could be adapted for future possible clinical use to fine-tune treatment parameters based on differences in patient ocular tissue geometries, which can differ by age, disease indication and other parameters that affect scleral photocrosslinking [41,42].

## 2. Methods

### 2.1. Model structure

Modelling was based on a computational model developed by Gerberich *et al*. [21] for the rat eye that used MATLAB (MathWorks, Natick, MA) and the ode15s ordinary differential equation solver function. Briefly, the model calculated the simultaneous anterior diffusion of MB from the injection reservoir, light penetration into tissue and kinetics of reactions to crosslink choroidal and scleral collagen. The concentrations over time and space between the choroid and Tenon’s capsule were calculated by assuming one-dimensional (1D) diffusion through these tissues. The model was structured around a 1D tissue segment from Tenon’s capsule to choroid, which was simulated by dividing the tissue into *n* finite-element segments. The same number of tissue segments or nodes, *n*, was used for all species, although the absolute length of the tissue segments differed between species (i.e. rat tissue length was much less than minipig or human), as shown in electronic supplementary material, table S2. Seven nodes were used in Tenon’s capsule, 28 in sclera and 12 in choroid. We found that crosslinking calculations were similar when 36 nodes, rather than 12, were used in the minipig and human choroid (electronic supplementary material, table S2). Thus, 12 choroid nodes were used for the remainder of studies for consistency and to avoid longer computing times. Diffusion of MB and O_2_ species was modelled using Fick’s Second Law, which was discretized as described in our prior modelling work [21]. Light propagation was modelled by the Beer–Lambert Law, which was discretized and used to calculate light intensity, as described previously [21].

### 2.2. Model parameters

The model was adapted for simulating minipig and human tissue by changing tissue parameters (tissue lengths, amino acid and melanin concentrations and tissue densities). We expected that some model parameters should be approximately constant among rat, minipig and humas eyes, and therefore used values previously employed to model the treatment in rats (table 2) [21]. However, other parameters needed to be separately specified for each of the three species considered in this study (table 1).

**Table 2.** Model parameters assumed constant among rats, minipigs and humans.^a^

parameter (units)	description	value
*t (*s)	treatment time	1800
*I* (mW cm^−2^)	light intensity	424
*C_0_*_MB_ (mM)	MB injection concentration	3
λ (nm)	light wavelength	660
$\Phi_{Lumen}$	luminal fraction of choroid	5.1 × 10^−1^
$\Phi_{Fundus}$	fraction of light reflected by retinal pigment epithelium	9.6 × 10^−2^
$\Phi_{MBT}$	MB triplet quantum yield	5.2 × 10^−1^
$\varepsilon_{MBM}$ (M^−1 ^cm^−1^)	molar absorptivity of MB monomer	7.33 × 10^4^
$\varepsilon_{MBD}$ (M^-1 ^cm^−1^)	molar absorptivity of MB dimer	3.53 × 10^4^
$\varepsilon_{Mel}$ (M^-1 ^cm^−1^)	molar absorptivity of melanin	646
*D*_MB Reservoir_ (cm^2^ s^−1^)	diffusion constant of MB in injection reservoir	4.60 × 10^−6^
*D*_MB Tissue_ (cm^2^ s^−1^)	diffusion constant of MB in tissue	3.16 × 10^−7^
*D_O2_*_Tissue_ (cm^2^ s^−1^)	diffusion constant of O_2_ in tissue	6.00 × 10^−6^
*K_D_* (M^−1^)	MB dimerization constant	1.0665 × 10^4^
*k_1_* (M^−1 ^s^−1^)	MB dimer formation	3.02 × 10^8^
*k_2_*	MB dimer dissociation	*k_1_ × K_D_^−1^*
*k_3_* (s^−1^)	MB triplet physical quenching by solvent	1.3 × 10^4^
*k_4_* (M^−1 ^s^−1^)	MB triplet physical quenching by:	MB monomer: 4.1 × 10^7^ Met: 1 × 10^8^ Cys: 1.92 × 10^9^
*k_5_* (M^−1 ^s^−1^)	MB triplet chemical quenching by amino acids:	His: 2 × 10^6^Trp: 6 × 10^8^Tyr: 2.96 × 10^7^
*k_7_* (M^−1 ^s^−1^)	leuco MB formation from oxidation of reduced MB by ^3^O_2_	89
*k_8_* (M^−1 ^s^−1^)	crosslink formation from amino acid radical (type I pathway)	4 × 10^6^
*k_9_* (M^−1 ^s^−1^)	^1^O_2_ generation from MB triplet	2.6 × 10^9^
*k_10_* (s^−1^)	^1^O_2_ quenching by solvent	2.56 × 10^5^
*k_11_* (M^−1 ^s^−1^)	^1^O_2_ physical quenching by amino acids:	Trp: 2.1 × 10^7^ Tyr: 2.7 × 10^7^Cys: 4.2 × 10^7^
*k_13_* (M^−1^s^−1^)	oxidized amino acid generation from ^1^O_2_ chemical quenching by amino acids:	Met: 1.6 × 10^7^Cys: 8.9 × 10^6^
*k_14_* (M^-1 ^s^-1^)	crosslink formation from ^1^O_2_ chemical quenching by amino acids (type II pathway):	His: 1 × 10^8^ Trp: 3 × 10^7^ Tyr: 8 × 10^6^

^a^ Values obtained from prior model analysis [21].

O_2_ concentrations at values other than 21 or 100% inspired O_2_ for each species’ retrobulbar and choroidal O_2_ were calculated by Henry’s Law combined with linear interpolation between known concentrations at 0, 21 and 100% (if available in literature) inspired O_2_ (electronic supplementary material, table S3). O_2_ concentration at 0% inspired O_2_ was assumed to be 0 M.

### 2.3. Tissue volume available for crosslinking

Scleral crosslinking for glaucoma treatment is proposed to be targeted to a region of sclera that rings the optic disc [10,17]. We calculated the volume (*V*) of a hollow spherical frustum comprising the peripapillary tissue targeted for selective photocrosslinking by assuming the eye to be a perfect sphere in all species (electronic supplementary material, §S1).

### 2.4. Methylene blue injection reservoir

Injection of MB in the eye is performed in the retrobulbar space, which creates an MB injection reservoir with a depth (i.e. distance occupied by MB reservoir in retrobulbar tissues behind Tenon’s capsule) that was calculated by assuming the retrobulbar injection reservoir could be approximated as a cylinder of the same radius as the eye [21]. A 100 µl volume of injected MB was modelled for all species, resulting in different injection reservoir depths across species (table 1). The full calculations are shown in electronic supplementary material, § S2.

## 3. Results

### 3.1. Crosslinking extent, rate and spatial distribution between species

We compared the extent, rate and spatial distribution of scleral crosslinking in rat, minipig and human as a function of treatment conditions.

The predicted absolute number of crosslinks is similar across species when compared at the same baseline treatment conditions (table 3). However, the percentage of crosslinking sites used was much bigger in rats (6.57%) compared to minipigs or humans (0.070 or 0.039%, respectively), because the total number of possible crosslink sites is so much smaller in rats, and similar to each other in minipigs and humans. When crosslink concentrations on a molar basis are compared between species, the concentrations in minipig (5.75 × 10^−5^ M) and human (3.84 × 10^−5^ M) are again much smaller than in rat (5.24 × 10^−3^ M).

**Table 3.** Comparison of scleral crosslinking among rat, minipig and human eyes.^a^

	rat	minipig	human
total crosslinks (mol)	5.39 × 10^−10^	3.75 × 10^−10^	5.22 × 10^−10^
tissue volume^b^ (cm^3^)	1.03 × 10^−4^	6.53 × 10^−3^	1.36 × 10^−2^
total crosslink concentration (M)	5.24 × 10^−3^	5.75 × 10^−5^	3.84 × 10^−5^
crosslink sites possible (M)	7.97 × 10^−2^	8.20 × 10^−2^	9.87 × 10^−2^
crosslink site utilization (% crosslink sites possible)	6.57	7.00 × 10^−2^	3.90 × 10^−2^

^a^ Crosslinking was determined after 30 min treatment time at 21% inspired O_2_, 424 mW cm^−2^ laser fluence, 3 mM injected MB. Total crosslinks were averaged over tissue node thickness.

^b^ Based on volume of a hollow spherical frustum (see electronic supplementary material, §S1).

**Figure 2.**
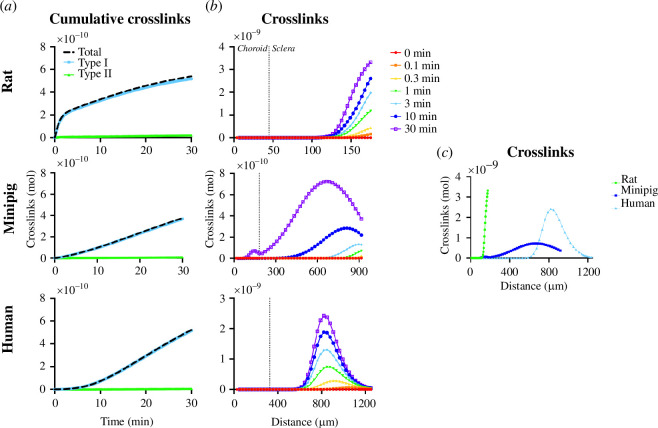
Scleral crosslinking as a function of time and position in rats, minipigs and humans. (*a*) Cumulative total crosslinks versus time as a function of reaction pathway types. Total crosslinks were averaged over tissue node thickness. (*b*) Crosslinks versus distance from the retinal pigment epithelium (RPE) as a function of time. The dotted line denotes the choroidal–scleral boundary. (*c*) Crosslinks versus distance from the RPE as a function of species after 30 min. Crosslinking was carried out for a 30-min treatment time at 21% inspired O_2_, 424 mW cm^−2^ laser fluence, 3 mM injected MB. Rat data are reproduced from Gerberich *et al*. [21].

When examining the kinetics of crosslinking, there is a rapid increase in crosslinks during the first 5 min in rat, which is followed by a slower crosslinking rate (figure 2*a*). In contrast, crosslinking in the minipig occurs at a relatively steady rate throughout the 30-min period, and crosslinking in the human eye exhibits a lag time for the first 5–10 min. We might interpret these kinetic data by noting that crosslinking occurs only in the presence of light, collagen, O_2_ and MB. Because tissue thickness is so much shorter in the rat eye, MB may be able to diffuse into the region of light penetration right away, allowing immediate and extensive crosslinking. In the minipig, the MB diffusional distance is longer, and in the human eye, it is longer still (figure 1), which results in a lag time until sufficient MB can diffuse into the illuminated tissue. This hypothesis is further addressed below.

While tissue length may explain this initial behaviour, the decrease in crosslink formation rate in the rat after 5 min, followed by roughly constant crosslinking after that, could be explained by the reactions reaching a steady state, where the rate of MB diffusion into illuminated tissue becomes rate limiting and is balanced with the rate of MB consumption. Human and minipig, by contrast, may be limited by MB diffusion throughout the process.

The distribution of crosslinks also differs among species (figure 2). Peak crosslinking concentration occurs in the middle of the sclera at 819 and 664 µm from the RPE (or 65 and 72% of the distance through the sclera) in human and minipig, respectively, while for rat, the peak in crosslinking is in the posterior sclera at 175 µm (100% of the distance through the sclera) next to the MB injection reservoir (i.e. which is located just approx. 200 µm from RPE, owing to the thin length of rat choroid and sclera). For all three species, crosslinking starts in the posterior sclera (i.e. near the MB reservoir) and then moves anteriorly over time. In humans, the crosslinking peak reaches the centre of the sclera faster than that for the minipig, possibly because of less deep light penetration across the thick human choroid and into the sclera (figure 1). The significance of these differences in spatial distribution is not clear, because it is not yet known how spatial distribution of crosslinks would affect scleral stiffening outcomes.

We also determined the contribution of types I and II reaction mechanisms [43]. In type I reactions, triplet-state MB reacts directly with amino acids on the collagen molecule to form amino acid radicals, which then react with ground-state O_2_ triplet to form crosslinked amino acids. In type II reactions, the MB triplet reacts with ground-state O_2_ triplet to form O_2_ singlet, which then reacts with collagen amino acids to form crosslinks [21]. We found that for all three species, the type I pathway significantly dominates over type II (figure 2*a*). A possible explanation could be that in the type I pathway, all five of the amino acids are involved in crosslink formation pathway, compared to pathway type II, where only histidine, tyrosine and tryptophan participate in the productive pathway.

### 3.2. Mechanistic understanding of crosslinking between species

Photocrosslinking requires the combination of light, MB, O_2_ and collagen. Light enters sclera from the anterior side and loses intensity as it crosses RPE, choroid and sclera. MB enters sclera from the posterior side and decreases in concentration as it diffuses anteriorly. O_2_ comes mostly from the vasculature in choroid and rapidly diffuses posteriorly across sclera, although some O_2_ is introduced at the posterior side of the sclera as part of the MB injection. Collagen is present throughout the sclera. We hypothesize that light penetration and MB diffusion are the rate-limiting steps for collagen photocrosslinking. We further hypothesize that spatial distribution of crosslinks in the sclera is governed largely by light penetration depth, which is roughly independent of time, and that temporal distribution of crosslinks in the 30-min treatment time is determined mostly by extent of MB diffusion from the injection reservoir into the sclera.

Light penetration depends on melanin concentration (i.e. because melanin absorbs light) and tissue length across RPE, choroid and sclera. Rat and minipig choroid have approximately five times more melanin than in humans, while human sclera has approximately five times more melanin compared to minipig, but is similar to rat (table 1). Additionally, choroid thickness increases from rat to minipig (four times thicker than rat) to human (seven times thicker than rat). The combination of different tissue lengths and melanin concentrations is expected to cause different distributions of light transmitted to tissues in each species.

**Figure 3.**
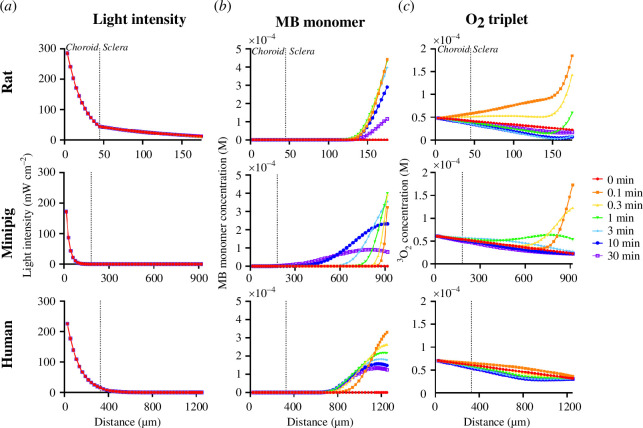
Spatial and temporal distribution of model parameters in rats, minipigs and humans including (*a*) light intensity, (*b*) MB monomer concentrations and (*c*) O_2_ triplet concentration. Vertical dotted line denotes choroidal-scleral boundary. Crosslinking was carried out for a 30-min treatment time at 21% inspired O_2_, 424 mW cm^−2^ laser fluence, 3 mM injected MB. Rat data are reproduced from Gerberich *et al*. [21].

Light intensity entering the sclera was highest in the rat, followed by human, then minipig (figure 3*a*). While rat choroid has high melanin content, its small thickness allowed the most light to pass. While human choroid is thick, its low melanin content allows more light to pass compared to the minipig, which has a thick choroid as well as a high melanin content. Once in the sclera, light intensity steadily decays as it penetrates posteriorly, with a less steep decay in minipig sclera owing to its lower melanin content (electronic supplementary material, figure S2).

In addition to light, MB monomers are required for photocrosslinking. Injected MB was assumed to contain monomers and dimers, which then diffuse to anteriorly across sclera from the injection site. In rats, the MB monomer spatial distribution remains concentrated in the posterior sclera throughout the 30 min (figure 3*b*). This is because light penetrates across the full sclera in rats, so that MB monomers are efficiently converted to triplet and reduced (leuco) MB as soon as they enter sclera. By contrast, in minipig and human, the MB diffusion front penetrates progressively further into the sclera over time. This can be explained by the low light intensity in the posterior sclera, which only partially converts MB monomers into triplet and leuco MB, thereby allowing more MB to diffuse anteriorly. These observations support the hypothesis MB monomer consumption (that leads to collagen crosslinking) depends largely on MB diffusion into the sclera.

Triplet O_2_ is a required reactant for either type I or II crosslinking scheme to occur. For all three species, there is a steady decay in O_2_ triplet concentration moving posteriorly from the source of O_2_ in the vasculature of the choroid (figure 3*c*). Immediately after injection, O_2_ triplet concentration becomes elevated in the posterior sclera, owing to additional O_2_ contributed from the MB injection reservoir. The effect is more dramatic in rat and minipig, but is attenuated in the human eye because MB injection reservoir length is shortest in human, which allows for fastest diffusion of O_2_ triplet towards the anterior tissue. Over time, the O_2_ concentration approaches its pre-injection distribution as the O_2_ reservoir at the posterior sclera is depleted.

To further study photocrosslinking, we also modelled the change in intermediate excited-state compounds such as O_2_ singlet, MB triplet and amino acid radicals. O_2_ singlet is formed in the type II reaction scheme when MB triplet converts O_2_ triplet to O_2_ singlet. MB triplet and O_2_ singlet react with collagen amino acids to form amino acid radicals, which in turn crosslink.

**Figure 4.**
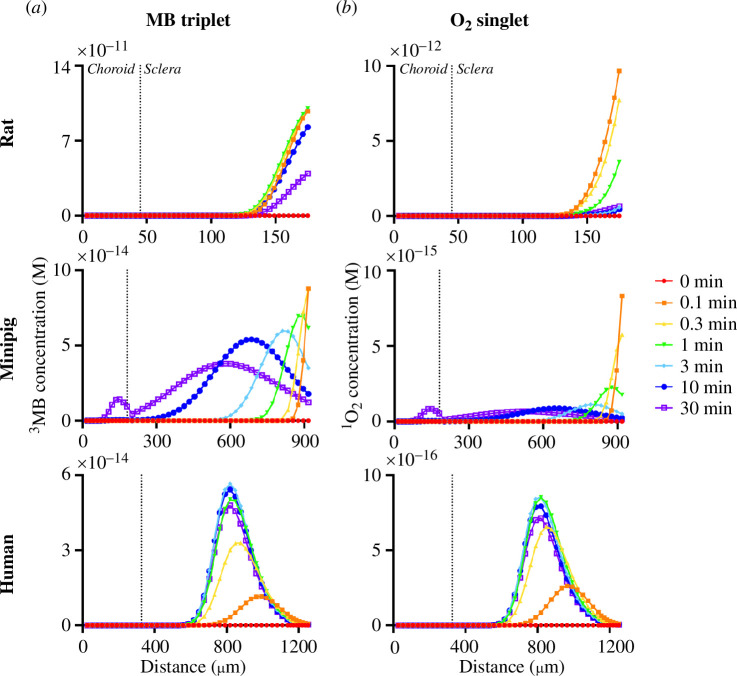
Spatial and temporal distribution of the concentration of excited-state intermediate compounds: (*a*) MB triplet and (*b*) O_2_ singlet. Vertical dotted line denotes the choroidal–scleral boundary. Crosslinking was carried out for a 30-min treatment time at 21% inspired O_2_, 424 mW cm^−2^ laser fluence, 3 mM injected MB. Rat data are reproduced from Gerberich *et al*. [21].

For rat, MB triplet (figure 4*a*), O_2_ singlet (figure 4*b*) and amino acid radicals (electronic supplementary material, figure S3) all have peaks that are concentrated towards the posterior edge of the sclera. The concentration of MB triplet and O_2_ singlet starts high and then decreases over time, but the peaks in the concentrations at each time remain in the same spatial location, and are spatially similar to rat MB monomer profiles (figure 3*b*). This spatial similarity between MB triplet, O_2_ singlet and MB monomer profiles over time indicates that conversion of MB monomer to MB triplet, which then converts O_2_ triplet to O_2_ singlet, both occur relatively faster than the unreacted species can anteriorly diffuse. This further supports the hypothesis that rat has fewer diffusional limitations owing to shorter tissue length. In contrast, amino acid radicals are formed more slowly, rising to a peak concentration for the first 3 min and then decreasing in concentration as crosslinks are formed.

In minipigs and humans, MB monomer concentration distributes anteriorly into the sclera (figure 3*b*), MB triplet concentration distribution extends even further anteriorly (figure 4*a*) and O_2_ singlet concentration distribution extends slightly more anterior still (figure 4*b*). This shift in distribution can be explained by a threshold in light intensity to drive the reactions, where MB monomer achieves a high concentration posteriorly, but then is converted to MB triplet (which further drives formation of O_2_ singlet) upon diffusion anteriorly into a region with higher light intensity. Amino acid radical distribution again shows a time lag in concentration (indicating slower kinetics) and has a distribution more like MB triplet distribution than O_2_ singlet distribution (electronic supplementary material, figure S3), which is consistent with the type I reaction pathway dominating (figure 2*a*).

We also modelled the change in amino acid concentrations with time, which are depleted by reaction with the MB triplet and O_2_ singlet to form amino acid radicals or other degradation products (electronic supplementary material, figure S4). In the rat, cysteine reacted extensively (with 98% depletion at the posterior edge of sclera over the course of 30 min), and tryptophan and histidine were also significantly consumed (47.2 and 13.4%, respectively). Tyrosine and methionine exhibited very little consumption. In contrast, the decrease in amino acid concentration in minipig and human sclera was a tiny fraction of the total amino acids (electronic supplementary material, table S4). This is because the much lower MB triplet and O_2_ singlet production (owing to lower light penetration into sclera) in minipigs and humans resulted in less amino acid radical production, relative to the much greater total amount of amino acids in these larger tissues (only up to 2.12 × 10^−6^% and 1.74 × 10^−6^% amino acids converted to radicals in humans and minipigs, respectively, compared to up to 2.58 × 10^−4^% in rats).

**Figure 5.**
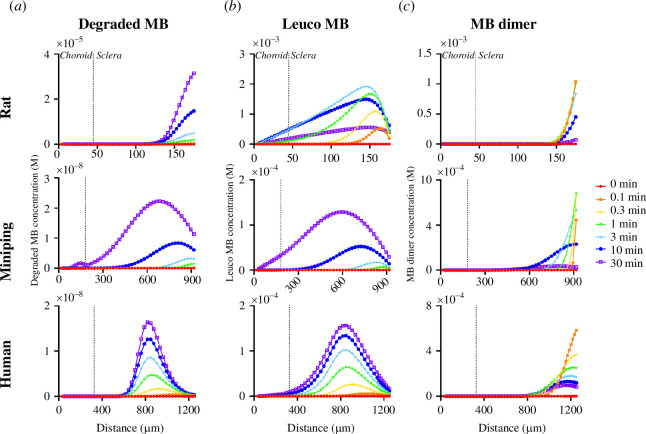
Spatial and temporal distribution of the concentration of unproductive compounds: (*a*) degraded MB, (*b*) leuco MB and (*c*) MB dimer. Vertical dotted line denotes choroidal–scleral boundary. Crosslinking was carried out for a 30-min treatment time at 21% inspired O_2_, 424 mW cm^−2^ laser fluence, 3 mM injected MB. Rat data are reproduced from Gerberich *et al*. [21].

Photocrosslinking is also limited by generation of unproductive MB species owing to side processes/reactions that form MB dimers, leuco-MB and degraded MB [21]. The concentration of these unproductive MB species is significantly higher than triplet MB (figure 5). This can be explained in part by the rapid reaction of triplet MB to leuco-MB in the course of productive crosslinking and is to be expected. Additionally, the rate constant for dimer formation is relatively large for MB and differs between photosensitizers (table 2).

#### 3.2.1. Optimal treatment conditions for maximal crosslinking

Injected MB concentration, laser fluence and per cent inspired O_2_ are the main conditions that could be adjusted in a clinical setting. Generally, we hypothesized that increasing these parameters would result in greater crosslinking. MB concentration and laser intensity may be increased relatively easily by adjusting the formulation and laser controls, respectively. Increasing inspired O_2_ above 21% atmospheric partial pressure requires administration of pure O_2_ (100% partial pressure). Even greater partial pressure can be achieved by increasing inspired air pressure. For example, 300% inspired O_2_ represents breathing pure O_2_ at a pressure three times greater than atmospheric pressure. These conditions could be achieved through the use of hyperbaric chambers already used for treatment of emergency and chronic conditions with compressed O_2_, usually in the range of 2.0–2.5 atm [44].

#### 3.2.2. Effect of injected methylene blue concentration

**Figure 6.**
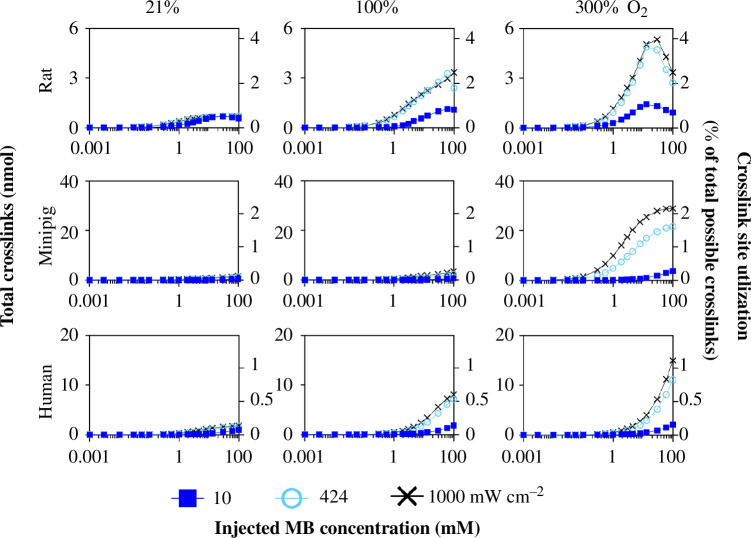
Scleral crosslinking as a function of MB concentration at varied laser fluence and inspired per cent O_2_ in rats, minipigs and humans. In each graph, crosslinks are shown as a function of MB concentration at three different laser fluence values (10, 424 and 1000 mW cm^−2^). These graphs are shown at three different per cent inspired O_2_ values (21, 100 and 300%) for each species. Rat data at 21% and 100% inspired O_2_ for 424 mW cm^−2^ are reproduced from Gerberich *et al*. [21].

We first examined the effect of increasing injected MB concentration and found that there are three regimes (figure 6). At least two of these regimes are qualitatively present for all three species and at all per cent inspired O_2_ and laser intensity conditions, although their quantitative values vary. At low MB concentration (<0.1 mM), there is very little crosslinking, suggesting insufficient MB to drive crosslinking reactions. At moderate MB concentration, crosslinking increases with increasing MB concentration, especially above a value of 0.1–1 mM MB, which indicates that MB reaches a sufficient level for crosslinking to occur and is a limiting factor in the crosslinking reaction. At high MB concentration (>13–30 mM), and seen most notably in the rat, crosslinking reaches a plateau or peak and then decreases with increasing MB concentration. We hypothesize that increasing MB concentration to still higher values would similarly show the third regime of decreasing crosslinking at all conditions.

The observation that crosslinking in rats increases and then decreases with increasing MB concentration suggests competing factors. At moderate MB concentrations, productive effects of MB on crosslinking dominate, but at higher MB concentrations detrimental effects, such as competing side reactions of MB, may dominate. More specifically, MB monomers and dimers can quench triplet MB [45], and dimers can absorb light unproductively. Both MB quenching and dimerization are unproductive to photoexcitation and crosslinking pathways since they consume reactive MB and O_2_ species and make monomeric MB unavailable for photoexcitation.

MB dimerization rate increases as MB monomer concentration increases because dimerization reaction rate is second order in MB monomer concentration, unlike crosslinking reactions, which are first order. The increased dimerization rate would then decrease the amount of MB monomer available to participate in crosslinking reaction pathways by a power of two. In rats at optimal conditions (30 mM MB, 300% inspired O_2,_ 1000 mW cm^−2^ laser intensity), dimers comprise 47% of MB species present at 10 min (6.35 times the monomer concentration at 10 min) and when MB concentration is increased to the third regime where crosslinking decreased at higher MB concentrations (100 mM MB, 300% inspired O_2_, 1000 mW cm^−2^ laser intensity), dimers comprise 85% of MB species (12.3 times monomer concentration). Crosslinks at the optimal conditions reach a maximum plateau in the choroid to the anterior sclera (until 108 µm through the RPE), while crosslinks in the third regime have the same profile in the choroid up to a shorter distance through the anterior sclera (up to 67 µm through RPE) (electronic supplementary material, figure S5). This maximum plateau of crosslinking coupled with the complete depletion of amino acids at similar spatial locations (electronic supplementary material, figure S6) suggests that almost all crosslinking sites in the choroid and anterior sclera were used. When MB concentration is 100 mM, there is more dimer that has diffused from the injection reservoir across the sclera (electronic supplementary material, figure S7) compared to at 30 mM MB; this limits crosslinking from the posterior side, resulting in maximum crosslinking stopping at just 67 µm from the RPE (for 100 mM MB), rather than at 108 µm (for 30 mM MB). There is a balance between more crosslinking when MB concentration is high and crosslinking becoming limited by the corresponding higher concentration of MB dimers diffusing from the injection reservoir.

#### 3.2.3. Effect of laser fluence

**Figure 7.**
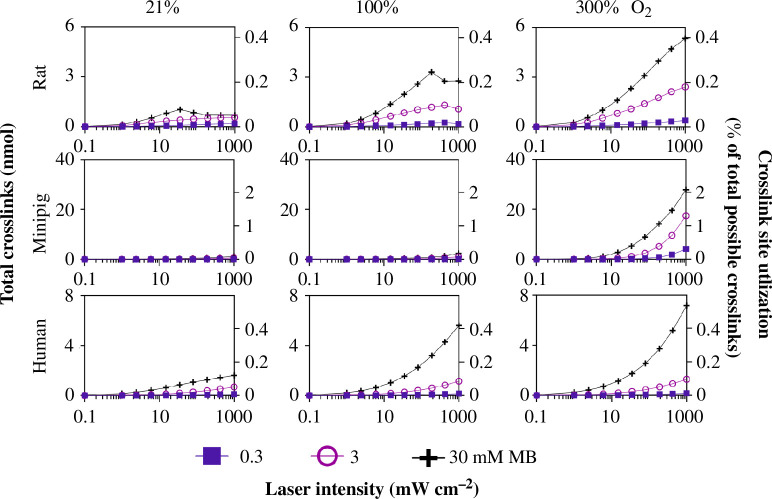
Scleral crosslinking as a function of laser intensity at varied MB concentration and inspired per cent O_2_ in rats, minipigs and humans. In each graph, crosslinks are shown as a function of laser intensity at three different MB concentrations (0.3, 3 and 30 mM). These graphs are shown at three different per cent inspired O_2_ values (21, 100 and 300%) for each species. These graphs contain the same data as in figure 6 but are replotted to highlight different effects on crosslinking. Rat data at 21 and 100% inspired O_2_ for 0.3, 3 and 10 mM MB are reproduced from Gerberich *et al*. [21].

We examined the effects of laser fluence on crosslinking, where we again see three regimes, with little crosslinking at low laser intensity (1–10 mW cm^−2^), increasing crosslinking with increasing laser intensity at moderate intensity values (generally < 100 mW cm^−2^), and evidence for decreasing crosslinking with increasing laser intensity at high-intensity values (generally > 100 mW cm^−2^), especially in the rat for 30 mM MB (figure 7). We hypothesize that increasing laser intensity to still higher values would similarly show the third regime of decreasing crosslinking at all conditions. These findings might be explained by the need for laser energy to drive the crosslinking reaction at moderate laser intensity values, while at high intensities and MB concentration but low oxygen concentrations, the oxygen may be depleted before reaching target crosslinking sites [46].

#### 3.2.4. Effect of percent inspired oxygen

**Figure 8.**
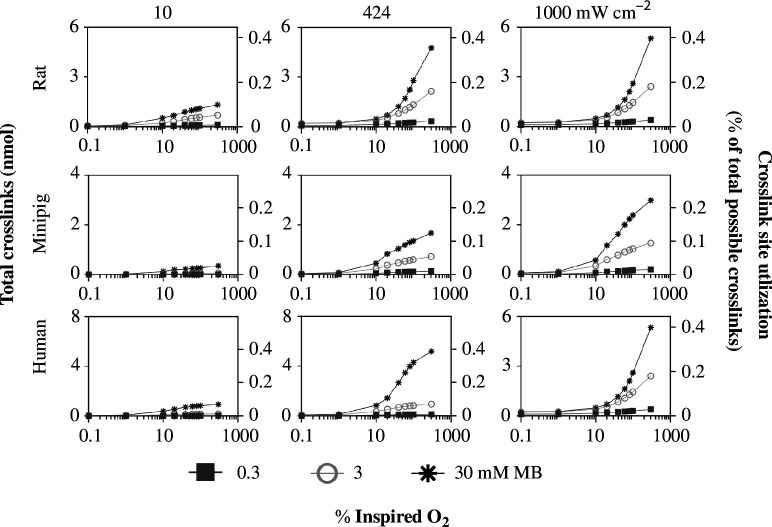
Scleral crosslinking as a function of per cent inspired O_2_ at varied MB concentration and laser intensity O_2_ in rats, minipigs and humans. In each graph, crosslinks are shown as a function of per cent inspired O_2_ at three different MB concentrations (0.3, 3 and 30 mM). These graphs are shown at three different laser intensity values 10, 424 and 1000 mW cm^−2^) for each species. These graphs contain the same data as in figures 6 and 7 but are replotted to highlight different effects on crosslinking.

Crosslinking increases monotonically with O_2_ over the modelled ranges (figure 8). This is consistent with the understanding that triplet O_2_ is needed for the crosslinking reaction. However, we do not see a third regime with decreasing crosslinking at high per cent inspired O_2_, perhaps because the O_2_ is not involved in the triplet MB quenching or MB dimerization reactions associated with the third regime seen above.

In summary, this analysis indicates that there can be optimal values of MB concentration (figure 6) and laser intensity (figure 7), and that increasing per cent inspired O_2_ is always beneficial to crosslinking (figure 8). Over the range of parameters tested, we have identified the optimal combination of treatment parameters for each species that maximizes the number of crosslinks formed (table 4). Maximum laser intensity, percent inspired O_2_ and injected MB concentration led to maximum crosslinking for all species over the range of parameters tested, except for rat, where 30 mM was the optimal injected MB concentration.

**Table 4.** Optimal treatment parameters for maximum crosslinking.

	rat	minipig	human
total crosslinks (mol)	5.33 × 10^−9^	2.89 × 10^−8^	1.50 × 10^−8^
crosslink site utilization (% crosslink sites possible)	64.9	5.41	1.11
laser intensity (mW cm^−2^)	1000	1000	1000
% inspired O_2_	300	300	300
injected MB concentration (mM)	30	100	100

At baseline crosslinking conditions initially considered (table 3), the total number of crosslinks is similar for each species and the total crosslink site utilization is very low. In contrast, at the optimal crosslinking conditions (table 4), the minipig has the highest number of crosslinks and the rat has the least. The low number of crosslinks in the rat can be explained by the much smaller number of available crosslink sites in the thin rat sclera and very high crosslink site utilization in the rat (i.e. available crosslink sites become rate limiting). The highest number of crosslinks in the minipig can be explained by the much greater number of possible crosslink sites compared to rats and the shorter tissue length scale that provides lesser MB diffusion and light penetration limitations compared to humans.

## 4. Discussion

### 4.1. Balancing optimal treatment conditions with safety

Our mechanistic understanding of crosslinking across the different species shows how increasing tissue length leads to greater barriers in MB diffusion and in light penetration depth, which leads to temporal and spatial limitations in crosslinking, respectively. It is evident that the increased tissue dimensions are the main limitation to crosslinking in larger species, since amino acid concentrations are of similar magnitude among the three species. In minipig and human, there is a threshold tissue depth where crosslinks are formed in the mid-scleral tissue, since light penetration depth and extent of MB monomer diffusion must be balanced for significant reaction to occur. It is unknown if this difference in spatial crosslink distribution in the mid-sclera in minipig and human would give different biomechanical outcomes from the rat, which has crosslinks in the posterior sclera. Additionally, owing to a lack of experimental data, it is unknown how many crosslinks are required in minipig and human to achieve a change in scleral stiffening similar to that achieved in rat [10,21]. It is expected that a different extent of crosslink site utilization may be required between species since previous studies have found that human scleral tissue is inherently stiffer than porcine and that riboflavin scleral photocrosslinking did not change biomechanical properties in *ex vivo* human eyes as much as in rabbit or porcine eyes [14].

The crosslinking limitations in larger species can be improved by increasing the per cent inspired O_2_, laser fluence, and injected MB concentration (although this may become detrimental at very high MB concentration, as seen in the rat). There are also lower thresholds of injected MB concentration, percent inspired O_2_ and laser fluence below which very little crosslinking occurs in each species.

Although increasing these three treatment parameters (per cent inspired O_2_, laser intensity, injected MB concentration) can improve crosslinking, there are safety limitations that must be balanced. Increasing laser intensity has the risk of adverse side effects, since exposure to visible light can cause damage by focusing radiation to the retina and from heat generation [47]. The maximum permissible dose of 660 nm light to human eyes for 30 min is 211 mW cm^−2^ [10], indicating that localized light damage is possible at the higher fluences examined in this study and associated side effects would need to be assessed. When increasing MB concentration, the treatment must remain within the limit for safe MB dose [48]; in this study, all MB concentrations would be in this range (<2 mg kg^−1^) [49] for minipigs and humans, but the higher MB concentrations would be above this limit for rats. Considering the per cent inspired O_2_, current hyperbaric chambers operate between 2 and 2.5 times atmospheric pressure [44] and the highest per cent inspired O_2_ modelled (300%) is just above this range. Future studies will need to factor safety more rigorously into the treatment parameter optimization process.

Additionally, when the number of crosslinks was maximized at optimal crosslinking conditions, some crosslinking also occurred in the choroid for rats and minipigs (electronic supplementary material, figure S5A). Crosslinking of the choroid could have negative consequences, for example, in myopic patients, since choroidal thinning and reduction of choroidal blood flow have been correlated with myopia progression [50]. However, it is unknown if crosslinking the choroid in our study would lead to choroidal thinning or reduced blood flow or have other possible adverse effects.

### 4.2. Model limitations

The model, originally developed for the rat eye, has several limitations for predicting scleral crosslinking in larger species. We used the same number of tissue slices for each species, which resulted in species with larger tissue length scales having tissue slices each of greater length. Larger tissue slices could mean less precise calculation across larger tissue lengths, but we found that decreasing the tissue slice thickness by increasing the number of tissue slices by threefold did not significantly change crosslinking outcome predictions (electronic supplementary material, table S2). The number of tissue slices could have been increased for larger tissue lengths to make slice thickness the same between species, but this would have significantly increased computing times for the minipigs and humans.

The same volume (100 µl) of injected MB was modelled across all species, which means that MB injection reservoir length decreases with increasing eye radius. It is unknown if increasing the volume of MB injected to scale with the increasing eye size would have given different results.

Also, the assumption of a perfect cylinder for the retrobulbar MB injection reservoir and a perfect hollow spherical frustum for the tissue volume available for crosslinking are simplified geometries. Retrobulbar space is actually more irregular in shape, and eyes, especially those of glaucomatous or myopic patients, are not perfect spheres [51].

Finally, the parameter values summarized in tables 1 and 2 represent reasonable estimates from literature and calculation, but their accuracy could be improved, and variability among individuals should also be accounted for.

### 4.3. Future work

Future experimental studies should assess the accuracy of model predictions of scleral crosslinking in minipig and human eye made in this study. Additional studies could correlate the number of crosslinks needed for minipig and human eyes to achieve the same increase in scleral mechanical strength as seen in rat. It is unknown if a similar extent of crosslinking is required in minipig or human sclera to achieve similar treatment outcomes that were achieved in rat [10] or if those levels of scleral stiffening will be effective to treat glaucoma [17]. Additionally, experiments should be done to determine if spatial crosslinking distribution in minipig and human being in the centre of the sclera gives different biomechanical outcomes than in rat, which has crosslinking in the posterior sclera. The current study also serves as a foundation for enabling future *in vivo* studies to test the hypothesis that selective peripapillary scleral crosslinking helps prevent glaucomatous damage to the optic nerve.

Future computational studies could model alternative photosensitizers/light wavelengths and longer treatment times, possibly using a lower light intensity. Longer treatment time could allow for MB monomer diffusion to have more time for better spatial distribution in the sclera before reacting with light to form crosslinks. Lower light intensity could enable staying within the intensity limits for safe laser exposure. Similarly, pulsed light exposure or waiting to apply light until after the MB monomer diffuses across the sclera could be modelled and could yield better spatial distribution of crosslinks. The use of sub-Tenon injection of MB, rather than retrobulbar, could also be modelled, since diffusion of MB from the sub-Tenon space could be an alternative to retrobulbar injection.

### 4.4. Conclusions

In summary, we show adaptation of a first-principles computational model with no fitted parameters for transpupillary peripapillary scleral photocrosslinking treatment in rats to predict crosslinking extent in minipig and human eyes. We present a mechanistic understanding of the photocrosslinking reactants and intermediates to determine the greatest limitations to crosslinking in all three species. Increasing tissue length when moving from rodent to larger animal and human eyes was shown to be the greatest barrier to achieving similar crosslinking extent in larger species, owing to limited anterior diffusion of MB from its posterior (retrobulbar) site of injection and limited posterior penetration of light from its anterior (transpupillary) site of illumination. Additionally, with the knowledge of crosslinking limitations in each species, we optimized three main treatment parameters (injected MB concentration, laser fluence and per cent inspired O_2_) to improve crosslinking extent. Overall, this study presents *de novo* predictions of photocrosslinking of sclera across three different species and presents strategies to control and optimize crosslinking in the context of anatomical and physiological parameters; MB, O_2_ and light penetration into tissues; and photocrosslinking reactions, including unproductive side reactions.

## Data Availability

Data are available in the article and the supplementary material [52].
